# Characterization of a Novel Insect-Induced Sesquiterpene Synthase GbTPS1 Based on the Transcriptome of *Gossypium barbadense* Feeding by Cotton Bollworm

**DOI:** 10.3389/fpls.2022.898541

**Published:** 2022-07-13

**Authors:** Hang Zhang, Enliang Liu, Xinzheng Huang, Junfeng Kou, Dong Teng, Beibei Lv, Xiaoqiang Han, Yongjun Zhang

**Affiliations:** ^1^Key Laboratory of Oasis Agricultural Pest Management and Plant Protection Resources Utilization, Xinjiang Uygur Autonomous Region, College of Agriculture, Shihezi University, Shihezi, China; ^2^State Key Laboratory for Biology of Plant Diseases and Insect Pests, Institute of Plant Protection, Chinese Academy of Agricultural Sciences, Beijing, China; ^3^Institute of Grain Crops, Xinjiang Academy of Agricultural Sciences, Urumqi, China; ^4^College of Plant Protection, China Agricultural University, Beijing, China; ^5^Institute of Plant Protection, Cangzhou Academy of Agriculture and Forestry Sciences, Cangzhou, China

**Keywords:** extra-long staple cotton, transcriptome sequencing, sesquiterpene biosynthesis, GbTPS1, β-elemene, *Helicoverpa armigera*, induced defense

## Abstract

When attacked by insect herbivores, plants initiate sophisticated defenses mediated by complex signaling networks and usually release a blend of functional volatiles such as terpenes against infestation. The extra-long staple cotton *Gossypium barbadense* cultivated worldwide as natural textile fiber crop is frequently exposed to a variety of herbivores, such as cotton bollworm *Helicoverpa armigera*. However, little is known about insect-induced transcriptional changes and molecular mechanisms underlying subsequent defense responses in *G. barbadense*. In the current study, transcriptome changes in *G. barbadense* infested with chewing *H. armigera* larvae were investigated, and we identified 5,629 differentially expressed genes (DEGs) in the infested cotton leaves compared with non-infested controls. *H. armigera* feeding triggered complex signaling networks in which almost all (88 out of 90) DEGs associated with the jasmonic acid (JA) pathway were upregulated, highlighting a central role for JA in the defense responses of *G. barbadense* against target insects. All DEGs involved in growth-related photosynthesis were downregulated, whereas most DEGs associated with defense-related transcript factors and volatile secondary metabolism were upregulated. It was noteworthy that a terpene synthase gene in the transcriptome data, *GbTPS1*, was strongly expressed in *H. armigera*-infested *G. barbadense* leaves. The upregulation of *GbTPS1* in qPCR analysis also suggested an important role for *GbTPS1* in herbivore-induced cotton defense. *In vitro* assays showed that recombinant GbTPS1 catalyzed farnesyl pyrophosphate and neryl diphosphate to produce three sesquiterpenes (selinene, α-gurjunene, and β-elemene) and one monoterpene (limonene), respectively. Moreover, these catalytic products of GbTPS1 were significantly elevated in *G. barbadense* leaves after *H. armigera* infestation, and elemene and limonene had repellent effects on *H. armigera* larvae in a dual-choice bioassay and increased larval mortality in a no-choice bioassay. These findings provide a valuable insight into understanding the transcriptional changes reprogramming herbivore-induced sesquiterpene biosynthesis in *G. barbadense* infested by *H. armigera*, which help elucidate the molecular mechanisms underlying plant defense against insect pests.

## Introduction

Of the two cultivated cotton species, the extra-long staple cotton *Gossypium barbadense* produces superior-quality fibers and is widely planted across the world. However, *G. barbadense* is frequently subject to attack by a wide range of insect pests such as aphids, mirid bugs, and cotton bollworm *Helicoverpa armigera* (Ayubov and Abdurakhmonov, 2018; Llandres et al., 2018). Cotton bollworm has caused considerable costs and yield losses as well as overuse of chemical pesticides (Li et al., 2020). In China, *Bacillus thuringiensis* (Bt)-transgenic cotton cultivars have been widely planted for managing serious lepidopteran pests including *H. armigera*, and provided efficient and lasting control of target pests. However, sole reliance on Bt-transgenic crops can lead to pest resistance to specific Bt proteins (Lu et al., 2022). Consequently, it is urgently needed to develop new environmentally friendly pest management strategies utilizing the plant’s own defense system.

Plants have evolved sophisticated, diverse systems against a wide array of insect herbivores herbivory that can be triggered by insect feeding, herbivore egg deposition and even insect walking (Wu and Baldwin, 2010; Turlings and Erb, 2018). The defense system in plants is initiated by a series of early signaling components such as Ca^2+^, reactive oxygen species, and MAP kinases, then a dynamic and complex transcriptional reprogramming is mediated by signaling networks including phytohormones and transcription factors (Erb and Reymond, 2019), which finally leads to the synthesis and accumulation of toxic chemicals (direct defenses) and release of diverse herbivore−induced plant volatiles that attract natural enemies (indirect defenses).

In response to insect infestation, cotton plants emit complex blends of terpene volatiles as an important part of the plant defense (Rodriguez-Saona et al., 2002; Degenhardt et al., 2011). It was found that *Gossypium hirsutum* immediately emits constitutive terpene volatiles such as α−pinene and (*E*)−β−caryophyllene in response to herbivores including *H. armigera*, *Spodoptera exigua*, and *Apolygus lucorum*, whereas several other terpenes including limonene, (*E*)−β−ocimene and linalool are synthesized *de novo* (Huang et al., 2018, 2022; Arce et al., 2021). These terpenes play crucial ecological roles in direct and indirect defenses in cotton plants. For instance, the homoterpene compound (3*E*)-4,8-dimethyl-1,3,7-non-atriene (DMNT) released from herbivore-induced *G. hirsutum* suppresses olfactory−triggered orientation of the moth *Spodoptera littoralis* to host plants and sex partners (Hatano et al., 2015). Similarly, a behavioral bioassay showed that DMNT and (3*R*)−linalool in volatile blends of *G. hirsutum* had repellent effects on females and males of *S. littoralis* (Borrero-Echeverry et al., 2015). *Apolygus lucorum*-induced cotton volatile blends dominated by terpenes significantly attracted the parasitoid *Peristenus spretus* (Huang et al., 2022). In addition, enhanced (*E*)-β-caryophyllene emission in transgenic *G. hirsutum* has a deterrent effect on *A. lucorum*, *Aphis gossypii*, and *H. armigera*, and significantly attracts the parasitoids *P. spretus* and *Aphidius gifuensis* (Zhang et al., 2020).

Volatile terpenes dominated by mono−, sesqui−, and diterpenes are synthesized by terpene synthases/cyclases (TPSs) using a few acyclic prenyl diphosphate precursors such as neryl diphosphate (NPP), geranyl diphosphate (GPP), farnesyl diphosphate (FPP), and geranylgeranyl diphosphate (GGPP) as substrates (Zhou and Pichersky, 2020). Based on phylogenetic relationships, plant TPS family fall into eight subfamilies (TPS-a to TPS-h). In angiosperms, TPS−a subfamily contains primarily sesquiterpene synthases, TPS−b comprises cyclic mono- and hemiterpene synthases, members of TPS−f produce diterpenes and TPS−g generally contains acyclic monoterpenes (Booth et al., 2020). The published genomic sequence data of *G. barbadense* (Liu et al., 2015; Hu et al., 2019; Wang et al., 2019) revealed that there are 115 putative *TPS* genes including 44 monoterpene, 59 sesquiterpene, and eight diterpene synthases. However, little is known about the defense roles of *TPSs* in *G. barbadense* against insects until now.

In the current study, transcriptional changes in *G. barbadense* after a 36-h infestation by *H. armigera* were investigated to identify key pathways involved in the activation of cotton plant defense and candidate terpene synthases. Furthermore, the sesquiterpene synthase gene *GbTPS1* was newly identified based on RNA-seq. And then the recombinantGbTPS1 was heterologously expressed in *Escherichia coli*. The catalytic products of the recombinant GbTPS1 were determined using an *in vitro* enzymatic assay and gas chromatography-mass spectrometry (GC-MS) analysis. What is more, the impacts of β-elemene and limonene on feeding behavior and growth of *H. armigera* larvae were also evaluated. These findings should contribute to developing markers for breeding new cotton cultivars with improved resistance to insect pests and further designing novel strategies for cotton pest control through the genetic modification of herbivore-induced terpene biosynthesis.

## Materials and Methods

### Plants and Insects

Cotton plants (*G. barbadense* cv. Hai7124) were sown in plastic pots (height, 14 cm; diameter, 16 cm). Seedlings were grown in a greenhouse compartment (29/25°C, 50 ± 10% RH, 16:8 L:D regime), and water was added every 2 days. All cotton plants were used in experiments at the 6–7 fully expanded true leaf stage (Huang et al., 2018). Laboratory colonies of cotton bollworm *H. armigera* were established from field−collected individuals. Larvae of *H. armigera* were reared on an artificial diet in a climate-controlled room (27 ± 2°C, 75 ± 10% RH, 14:10 L:D regime) (Liang et al., 2008). The 3rd instar larvae were used for all treatments.

### Plant Treatment

Each pot containing one cotton plant was placed in the greenhouse compartment. One *H. armigera* larva of was placed on a leaf of each cotton plant in a nylon mesh cage at 18:00 PM. After 36 h infestation, larvae were removed, and the damaged leaves were collected for RNA extraction. Non-infested plants in cages maintained under the same conditions were used as controls. For each treatment, three biological replicates were employed.

### RNA Extraction and RNA Sequencing

Total RNA was extracted using the easy-spin Total RNA Extraction Kit (Aidlab Biotechnologies Co., Ltd., Beijing, China). RNA quality was assessed using an Agilent 2100 Bioanalyzer (Agilent Technologies, Palo Alto, CA, United States). About 1 μg RNA per sample was used to construct a cDNA library using the NEBNext^®^ Ultra™ RNA Library Prep Kit, and then was sequenced on an Illumina Hiseq 2500 platform (Gene Denovo Biotechnology Co., Ltd., Guangzhou, China) using a PE150 strategy. Clean reads were mapped to *G. barbadense* genome (Hu et al., 2019) using Hisat2 software.

Gene expression levels were calculated based on fragments per kilobase of transcript per million fragments mapped (FPKM). Genes with log_2_(fold change) > 1 and false discovery rate < 0.05 were defined as differentially expressed genes (DEGs). The DEGs were functionally annotated using the Gene Ontology (GO) database and Blast2GO.^1^ DEGs were assigned to KEGG pathways using KOBAS 2.0 software (Xie et al., 2011).

### Cloning of *GbTPS1* and Heterologous Expression

In the transcriptome, *GbTPS1* (GB_D01G0996) was significantly expressed after *H. armigera* infestation. Subsequently, complete open reading frame (ORF) of *GbTPS1* was fully sequenced and inserted into the expression vector pET-30a (+). The plasmid was then inserted into *E. coli* strain BL21 (DE3) for heterologous expression. Freshly transformed *E. coli* cells were cultured in 500 mL LB with kanamycin (50 mg mL^–1^) at 37°C to an OD_600_ of 0.6. Then, 1 mM isopropyl 1-thio-β-D-galactopyranoside (IPTG) was added, and the cultures were incubated at 18°C for 20 h. Bacterial cells were pelleted by centrifugation at 8,000 × *g* for 10 min at 4°C and resuspended in 4 mL of extraction buffer (50 mM MOPS [pH 7.0], 5 mM MgCl_2_, 5 mM sodium ascorbate, 5 mM dithiothreitol, 0.5 mM phenylmethanesulfonyl fluoride and 10% [v/v] glycerol). Finally, the cells were disrupted by sonication, and the suspension was centrifuged at 14,000 × *g*.

### Enzymatic Activity Assay

Catalytic activities of the recombinant GbTPS1 were assayed as reported previously (Huang et al., 2018). In screw-capped 20-mL glass vials, 500 μL of *E. coli* crude extract, 10 μM of geranyl pyrophosphate (GPP), NPP, farnesyl pyrophosphate (FPP) or GGPP as the substrate, 10 mM MgCl_2_, 0.2 mM Na_2_WO_4_, 0.05 mM MnCl_2_ and 0.1 mM NaF, 10 mM MOPS (pH 7.0), 1 mM dithiothreitol and 10% (v/v glycerol). Samples were incubated for 1 h at 30°C. Solid phase microextraction (SPME, SAAB-57330U, Sigma-Aldrich, Shanghai, China) handle with a 65 μm polydimethylsiloxane (PDMS) fiber was inserted into the vial at a distance of 1 cm from the liquid surface and absorbed at 30°C for 30 min. Then the handle containing volatiles samples was inserted into a QP2010 SE gas GC-MS system (Shimadzu, Kyoto, Japan) equipped with an Rxi-5Sil column (30 m × 0.25 mm × 0.25 μm). The oven temperature was set at 40°C for 1 min, then raised 4°C/min to 130°C and held for 5 min, then increased at 10°C/min to 250°C and held for 5 min. Identity of (*R*)-(+)-limonene (CAS:5989-27-5) and β-elemene (CAS: 515-13-9) was confirmed by comparing retention times and mass spectra with those of the authentic standard (Sigma-Aldrich) under the same conditions. However, selinene and gurjunene are not commercially available, so we performed a comparison of mass spectra with those reported in the NIST17 library. The raw extracts from *E. coli* expressing empty vector pET-30a (+) were set as controls.

### Quantitative Real Time PCR Measurement

cDNA was synthesized from the total RNA using the FastQuant RT Kit as described above. The quantitative real time PCR (qPCR) analysis was carried out using SuperReal PreMix Plus (SYBR Green) reagent kit (TianGen, Beijing, China) (Huang et al., 2018). GhACT4 (accession number: AY305726) was used as an endogenous control (Supplementary Table 1). Three biological replicates were performed.

### Identification of Volatile Terpenes Emitted From Leaves of *Helicoverpa armigera*-Infested *Gossypium barbadense* Plants

The volatile terpenes emitted from *H. armigera*-infested or control *G. barbadense* were collected by headspace-SPME as described previously (Avellaneda et al., 2021). Briefly, cotton leaves were gently enclosed in 3 L polyethylene terephthalate containers. The headspace volatiles emitted from *G. barbadense* were collected for 4 h by SPME as described above. Ethyl nonanoate (17.2 mg, CAS: 123-29-5) was added as an internal standard. Then, the samples were analyzed using GC-MS as described above for enzymatic activity assay. Three biological replicates for each treatment were analyzed.

### Herbivore Bioassays

The choice behavior of *H. armigera* larvae to β-elemene or limonene at three different concentrations (0.1, 1, and 10 μg/μL) were assessed in plastic petri dishes (9 cm diameter) (Guo et al., 2021). A piece of 1 × 1 cm artificial diet containing 20 μL chemical samples was placed at one end of the petri dish, the control artificial diet containing n-hexane was placed at the other end. Fifty *H. armigera* larvae of third instar were tested in each compound and each concentration, and three biological replicates were analyzed. After 2 h, the choice of insects was recorded. Survival rate of *H. armigera* larvae on β-elemene or limonene at four different concentrations (0.05, 0.1, 1, and 10 μg/μL) on growth of *H. armigera* larvae was also investigated. Aliquots (20 μL) of β-elemene or limonene dissolved in hexane were added in artificial diet. Artificial diet containing n-hexane was used as control. One newly hatched larva per well (1.5 cm in diameter × 2 cm in height) were reared on 500 mg artificial diet in a 24-well plate and allowed to feed for 7 days. A total of 24 insects were tested in each compound and concentration, and five biological replicates were analyzed.

### Data Analyses

Data analyses were performed using SPSS Statistics software (version 17.0) (SPSS Inc., Chicago, IL, United States). Data are given as the mean ± standard error of the mean (SEM) and if needed, were transformed before analyses. Differences among treatment groups for target gene expression data and survival rate data were analyzed using a one-way analysis of variance (ANOVA) followed by Duncan’s multiple range test. In the behavioral assay, a chi-square test (50:50 distribution) was used to test for significant differences in larval choice between controls and treatments.

## Results

### Global Transcriptional Changes in *Gossypium barbadense* in Response to *Helicoverpa armigera* Infestation

Sequencing of the transcriptome from *G. barbadense* leaves generated 40,502,916–46,404,650 raw reads per sample, and the quality control steps generated 40,457,602–46,358,304 high quality reads. A PCA plot based on the transcriptomic data was shown in Supplementary Figure 1. Among these genes, 5,629 were associated with a cutoff of *q* ≤ 0.05 and log_2_(fold change) ≥ 1, and thus identified as DEGs. Among these DEGs, 2,460 were significantly upregulated after infestation, while 3,169 were downregulated (Supplementary Table 2).

**FIGURE 1:**
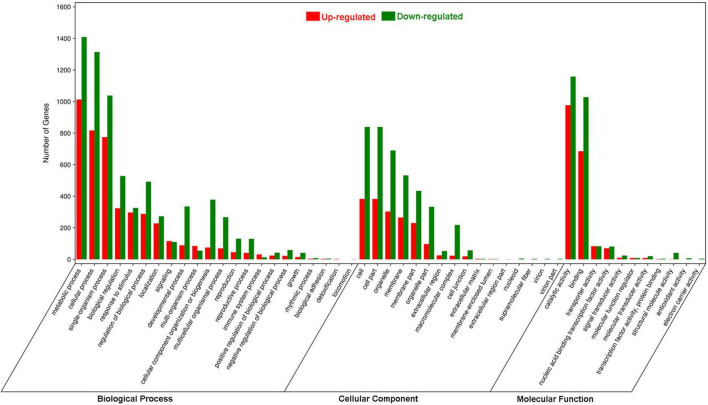
Gene Ontology enrichment analysis of differentially expressed genes (DEGs) in *Gossypium barbadense* leaves after *Helicoverpa armigera* infestation. The DEGs were judged as differentially expressed when log_2_| Ratio| ≥ 1 with FDR ≤ 0.05.

**FIGURE 2:**
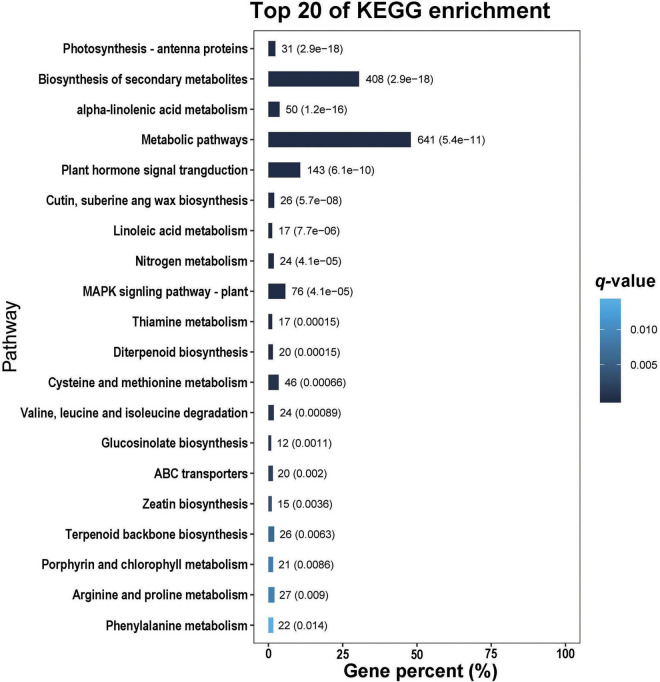
The top 20 pathways of differentially expressed genes (DEGs) identified in *Gossypium barbadense* leaves after *Helicoverpa armigera* infestation.

In the GO analysis, these genes were classified into biological process, cellular component and molecular function (Figure 1). In the biological process category, the top three terms were metabolic process (GO 0008152; 1013 up- and 1409 downregulated genes), cellular process (GO 0009987; 817 up- and 1314 downregulated genes), and single-organism process (GO 0044699; 775 up- and 1038 downregulated genes). In the category of cellular component, the most abundant were cell (GO 0005623; 383 up- and 839 downregulated genes) and cell part (GO 0044464; 383 up- and 839 downregulated genes), followed by organelle (GO 0043226; 303 up- and 690 downregulated genes), membrane (GO 0016020; 265 up- and 532 downregulated genes) and membrane part (GO 0044425; 230 up- and 434 downregulated genes). In the molecular function ontology, most DEGs were mapped to catalytic activity (GO 0003824; 977 up- and 1158 downregulated) and binding (GO 0005488; 685 up- and 1028 downregulated). To identify the key biological pathways activated by *H. armigera* infestation, the KEGG pathway analysis was conducted. Among the top 20 pathways (Figure 2), the top three pathways were metabolic pathways (641 genes), biosynthesis of secondary metabolites (408) and plant hormone signal transduction (143).

**FIGURE 3:**
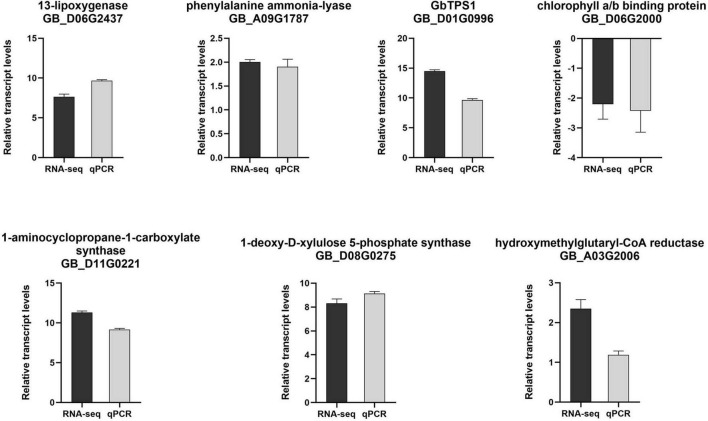
Log_2_(fold change) of selected genes in RNA-seq (black bar) and qPCR (gray bar) analysis.

To confirm the reliability and repeatability of the RNA-seq data, seven DEGs involved in JA, salicylic acid (SA), ethylene pathway, photosynthesis and terpene volatiles biosynthesis were evaluated using qPCR measurement. It was found that these DEGs in qPCR results were highly consistent with the data obtained from RNA-seq (Figure 3), indicating the reliability of RNA-seq data.

### Differentially Expressed Genes Involved in Phytohormones of *Gossypium barbadense* in Response to *Helicoverpa armigera* Infestation

In the present study, 51 genes involved in the JA biosynthesis pathway and 39 genes involved in the JA signaling pathway were differentially expressed in leaves of *G. barbadense* after *H. armigera* infestation, and most (88 of 90) of these DEGs were upregulated. In addition, most of the DEGs involved in ethylene (22 of 23), gibberellic acid (11 of 16), SA (10 of 15), and abscisic acid (35 of 54) were upregulated, whereas genes associated with brassinosteroids (19 of 25), cytokinin (16 of 30), and auxin (42 of 71) were mostly downregulated (Supplementary Table 3).

### Differentially Expressed Genes Involved in Transcription Factors

Of 344 genes for TFs that were differentially expressed, 200 were upregulated and 144 were downregulated after *H. armigera* infestation. Among these DEGs, most belonged to the gene family bHLH (33 up- and 43 downregulated), followed by ERF (56 up- and 12 downregulated genes), MYB (35 up- and 17 downregulated genes), and WRKY (24 up- and 6 downregulated) (Supplementary Table 4).

### Differentially Expressed Genes Involved in Primary and Secondary Metabolism

After *H. armigera* infestation, many genes involved in amino acid metabolism (113 up- and 48 downregulated), carbohydrate metabolism (123 up- and 106 downregulated), and nucleotide metabolism (28 up- and 26 downregulated) were differentially expressed. Notably, all 54 DEGs related to photosynthesis were downregulated (Supplementary Table 5).

Numerous genes contributing to plant defenses such as cytochrome P450 (CYP) (38 up- and 33 downregulated), glutathione *S*-transferases (GSTs) (5 up- and 6 downregulated), and UDP-dependent glycosyltransferases (UGTs) (20 up- and 6 downregulated) were differentially expressed (Supplementary Table 6). Additionally, several genes involved in volatile secondary metabolite pathways were also differentially expressed after the larvae infestation. Six of 8 DEGs involved in green leaf volatiles production and 34 of 49 DEGs for phenylpropanoid or benzenoid biosynthesis were upregulated (Supplementary Table 6). Most DEGs related to terpenoid volatile biosynthesis (39 of 41 DEGs) were upregulated. It is also important to note that all 13 *TPSs*, the genes for key enzymes involved in terpene biosynthesis, were upregulated (Supplementary Table 6).

### Sequence Analysis and Functional Characterization of GbTPS1

**FIGURE 4:**
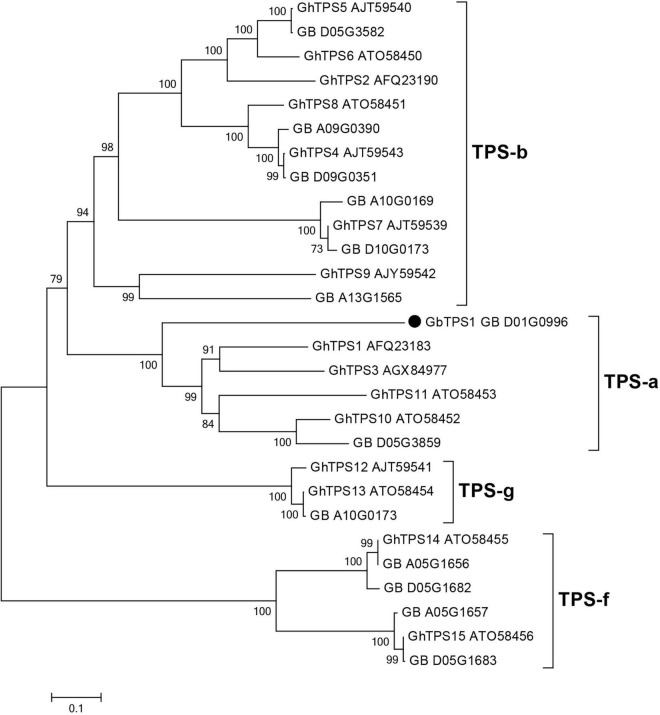
Phylogenetic tree of amino acid sequences of 13 TPSs identified in the transcriptome from *G. barbadense* leaves and previously characterized TPSs (GhTPS1-GhTPS15) from *Gossypium hirsutum*. The tree was constructed with the neighbor-joining method and 1,000 replications for bootstrapping.

The *GbTPS1* (GB_D01G0996) in transcriptome was strongly expressed after *H. armigera* infestation. This gene caught our attention and was selected for further functional characterization. *GbTPS1* has an ORF of 1653 bp, which encodes 551 amino acids with a predicted molecular mass of 64.18 kDa. Phylogenetic tree analysis showed that GbTPS1 was clustered within the TPS−a subfamily (Figure 4). The amino acid sequence of GbTPS1 has the typical DDxxD motif (NSE/DTE motif), the modified RR(x)_8_W motif in the N-terminal region, and the RxR motif located 35 amino acids upstream of the DDxxD motif (Huang et al., 2018).

**FIGURE 5:**
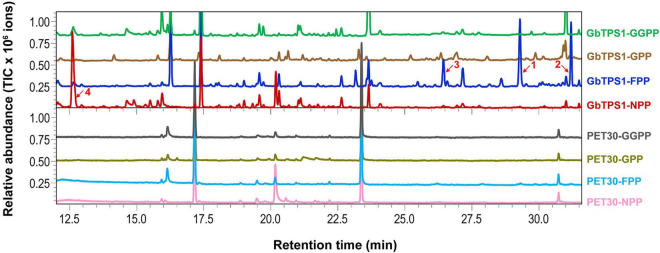
Gas chromatography-mass spectrometry analysis of the products catalyzed by recombinant GbTPS1. 1, selinene; 2, α-gurjunene; 3, β-elemene; 4, limonene. Other peaks are contamination.

The recombinant GbTPS1 were able to convert FPP into three sesquiterpenes selinene (48.20%), α-gurjunene (32.65%), and elemene (19.15%). When NPP was the substrate the catalytic product of recombinant GbTPS1 is a single limonene (Figure 5). No product was detected when GbTPS1 was incubated with GPP or GGPP as substrate. Meanwhile, cells expressing empty vector PET-30 did not produce target chemicals (Figure 5).

### Increased Emission of Terpenes From *Helicoverpa armigera*-Infested *Gossypium barbadense* Plants

**FIGURE 6:**
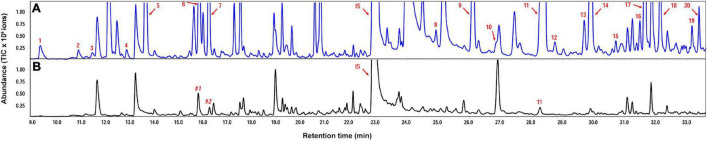
Representative chromatograms of volatile terpenes from *Gossypium barbadense* infested by *Helicoverpa armigera* **(A)** and from undamaged control plants **(B)**. Numbered compounds correspond to those in Table 1. IS, internal standard (ethyl nonanoate); #1, Non-anal; #2, 1,2,4,5-tetramethylbenzene.

*Helicoverpa armigera*-induced *G. barbadense* produced relatively high levels of volatile terpenes including six monoterpenes, 12 sesquiterpenes and two homoterpenes, whereas undamaged plants emitted only a trace amount of β−caryophyllene. Interestingly, *in vitro* products of GbTPS1–selinene, α-gurjunene, β-elemene, and limonene–significantly increased in the headspace volatiles from *H. armigera*-induced *G. barbadense* compared with those from undamaged plants (Figure 6; Table 1).

**TABLE 1:** Proportions (% of internal standard ethyl nonanoate) of volatile terpenes from *Gossypium barbadense* infested by *Helicoverpa armigera* and from undamaged control plants.

Compound	RT	Control	Herbivory
1. α-Pinene	9.29	nd	5.10 ± 2.43
2. β-Pinene	10.86	nd	4.04 ± 1.47
3. β-Myrcene	11.43	nd	1.71 ± 0.50*
4. Limonene	12.84	nd	3.92 ± 1.37*
5. (*E*)-β-Ocimene	13.62	nd	73.61 ± 27.42
6. Linalool	15.84	nd	18.99 ± 3.34**
7. DMNT	16.23	nd	24.70 ± 7.55*
8. Cubebene	24.93	nd	4.65 ± 0.83**
9. Copaene	26.13	nd	23.56 ± 4.59**
10. Elemene	26.87	nd	0.96 ± 0.07***
11. β-Caryophyllene	28.42	0.44 ± 0.19	85.20 ± 21.73*
12. Unknown sesquiterpene 1	28.75	nd	3.33 ± 1.77
13. Selinene	29.69	nd	2.58 ± 0.55*
14. α-Humulene	29.91	nd	32.70 ± 6.90**
15. Unknown sesquiterpene 2	30.71	nd	1.38 ± 0.19**
16. α-Gurjunene	31.48	nd	2.68 ± 0.22***
17. α-Farnesene	31.65	nd	27.33 ± 7.34*
18. δ-Cadinene	32.13	nd	10.53 ± 1.16**
19. Nerolidol	33.15	nd	1.68 ± 0.86
20. TMTT	33.38	nd	2.26 ± 0.25**

Numbered compounds correspond to those in Figure 6. Data were tested to evaluate differences using Student’s t-test (*P < 0.05; **P < 0.01; ***P < 0.001). DMNT, 4,8−dimethylnona−1,3,7−triene; TMTT, 4,8,12−trimethyltrideca−1,3,7,11−tetraene; RT, retention time; nd, not detected.

### The Impacts of β-Elemene and Limonene on Choice Behavior and Growth of *Helicoverpa armigera* Larvae

**FIGURE 7:**
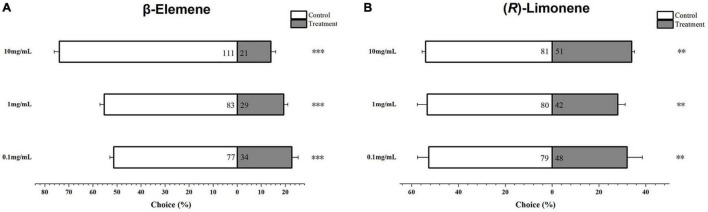
Behavioral preference of *Helicoverpa armigera* larvae to β-elemene **(A)** and limonene **(B)**. Data were tested for significant differences using χ^2^ analysis (**P < 0.01; ***P < 0.001).

**FIGURE 8:**
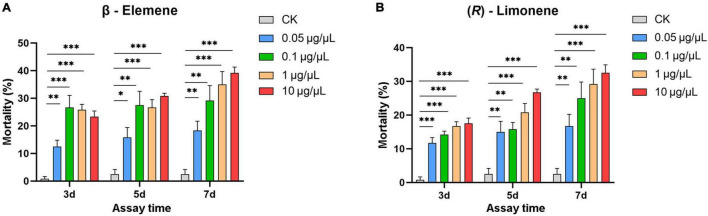
Larval mortality of *Helicoverpa armigera* caused by β-elemene **(A)** and limonene **(B)**. Data were tested to evaluate differences using Student’s *t*-test (*P < 0.05; **P < 0.01; ***P < 0.001).

*Helicoverpa armigera* larvae showed significantly preference for control diet over those containing β-elemene or limonene at the three concentrations (Figure 7; elemene: 0.1 μg/μL, χ^2^ = 16.658, *P* < 0.001; 1 μg/μL, χ^2^ = 26.036, *P* < 0.001; 10 μg/μL, χ^2^ = 61.364, *P* < 0.001; limonene: 0.1 μg/μL: χ^2^ = 7.567, *P* = 0.006; 1 μg/μL: χ^2^ = 11.836, *P* = 0.001; 10 μg/μL: χ^2^ = 6.818, *P* = 0.009). In the no-choice bioassay, the *H. armigera* larvae feeding on β-elemene and limonene at the four tested concentrations (0.05, 0.1, 1, and 10 μg/μL) showed significantly higher mortalities compared with those feeding on control diet (Figure 8).

## Discussion

*Gossypium barbadense* has excellent traits such as high resistance to biotic stresses. However, little is known about transcriptional changes in *G. barbadense* infested by herbivores. It is well known that herbivores often trigger complicated transcriptional responses and cross-talk between signal-transduction pathways such as JA, SA, ethylene, and abscisic acid in plants. The complex signaling networks link early signaling networks and downstream transcriptional reprogramming in plant defense responses against insect herbivore attack. Generally, JA pathway play a central role with synergistic or antagonistic effects of other phytohormone pathways, especially stress-related SA, ethylene, and abscisic acid pathways (Erb and Reymond, 2019). Consistently, our investigation showed that *H. armigera* larvae primarily activated JA signaling pathway. After *H. armigera* infestation, a total of 90 DEGs including 51 involved in the JA biosynthesis and 39 involved in the JA signaling were differentially expressed, of which almost all (88 DEGs) were upregulated (Supplementary Table 3). Notably, three DEGs (GB_A06G0079, GB_D06G0081, and GB_A05G1944) annotated as JA carboxyl methyltransferase (JMT) were upregulated in *H. armigera*-infested cotton plants. JMT catalyzes the formation of the volatile ester methyl jasmonate from JA and has been reported to be involved in herbivore-induced defense responses (Qi et al., 2016). In addition to JA pathway, a number of DEGs associated with other phytohormone pathways including ethylene, gibberellic acid, SA, abscisic acid, brassinosteroids, cytokinin, and auxin were also differentially expressed (Supplementary Table 3). These findings suggested that *H. armigera* infestation activated a complex signaling network in *G. barbadense*. Similar, in *G. hirsutum*, our group found that *H. armigera* infestation induced the expression of genes associated with JA and ET, and a complex regulatory pattern for genes involved in phytohormone pathways such as auxin, cytokinin, and brassinosteroids (Huang et al., 2015). Thus, a crosstalk between these signaling pathways might contribute to induced cotton defenses against *H. armigera*, and thereby lead to the synthesis and accumulation of secondary defense compounds.

Transcription factors that regulate expression levels of the target genes are known to be very important in activating downstream plant defense responses against insect herbivore attack. In this study, 344 TFs consisting mainly of bHLH (76 genes), ERF (68 genes), MYB (52 genes), and WRKY (30 genes) were differentially expressed in response to *H. armigera* infestation (Supplementary Table 4), which might contribute to the activation of different pathways of cotton plants to initiate sophisticated defense responses against *H. armigera*. TFs are involved in the biosynthesis of insect-induced volatile terpenes such as (*E*)-β-caryophyllene in *Arabidopsis thaliana* (Hong et al., 2012), (*E*)-geraniol and valencene in *Citrus sinensis* (Shen et al., 2016; Li et al., 2017), linalool in *Freesia hybrida* (Yang et al., 2020), α-farnesene in apple (Wang et al., 2020), (*E*)-β-farnesene and (*E*)-α-bergamotene in *Zea mays* (Li et al., 2015). In cotton, GaWRKY1 is involved in the accumulation of δ-cadinene, the essential precursor in the gossypol biosynthetic pathway (Xu et al., 2004). *H. armigera*-induced TFs, in particular those with an expression pattern correlated with the upregulation of 13 *TPSs* in transcriptome, likely play roles in the transcriptional regulation of the *H. armigera*-induced TPS genes.

Many genes associated with proteins or enzymes are involved in biosynthetic pathways of natural antibiotics. For example, CYP (71 genes), GSTs (11 genes), and UGTs (26 genes) were differentially expressed (Supplementary Table 6), suggesting possible roles in cotton defense against *H. armigera*. It is noteworthy that many genes associated with the biosynthetic pathways of volatile terpenoids (39 genes) and HPL-derived green leaf volatiles (6 genes) and shikimic acid−derived volatiles (34 genes) were upregulated after *H. armigera* infestation, whereas all 54 DEGs related to photosynthesis were downregulated (Supplementary Table 5), as resources were redirected toward the production of defense metabolites, as found for *G. hirsutum* after *H. armigera* infestation (Huang et al., 2015). Thus, the suppression of photosynthesis might be a general plant response to herbivore attack and contribute to reallocation of limited resources to hamper herbivore performance (Zhou et al., 2015; Huang et al., 2022).

In the transcriptomic data, all 13 TPSs were upregulated (Supplementary Table 6). Consistently, *H. armigera*-infested *G. barbadense* produced a high diversity of volatile terpenes (Figure 6). The upregulation of *GaTPS1* after *H. armigera* infestation was consistent with the elevated release of *in vitro* products of GaTPS1 including selinene, α-gurjunene, β-elemene, and limonene, which suggested that sesquiterpene synthase GbTPS1 is responsible for herbivore−induced terpene volatiles and contribute to anti-herbivore defenses in *G. barbadense*. The role of *GbTPS1* in terpene formation *in planta* is additionally strengthened by the fact that the ratios of selinene, α-gurjunene, and β-elemene (∼2.7:2.8:1) in the headspace volatiles from *H. armigera*-induced *G. barbadense* (Figure 6) appear similar to the product distributions of GbTPS1 (∼1.8:2.5:1) when FPP was the substrate (Figure 5). Moreover, insect bioassay data showed elemene and limonene had repellent effects against *H. armigera* larvae (Figure 7) and increased mortalities of *H. armigera* (Figure 8). These findings suggested that *GaTPS1* play a role in cotton plant direct defenses. Our findings not only enrich the understanding of terpenoid diversity in cotton plants, but also facilitate the discovery of new TPSs through transcriptome mining and homology-based searches.

In summary, the leaves of *G. barbadense* underwent extensive transcriptomic reprogramming that was regulated by complex phytohormonal signaling networks responding to *H. armigera* infestation. Almost all (88 out of 91) DEGs in the JA pathway were upregulated, highlighting that the *H. armigera* infestation mainly activated JA-mediated defense responses, thereby defense-related genes and pathways such as fatty acid-derived, shikimic acid−derived and terpenoid-related secondary metabolism were activated. The upregulation of *GbTPS1* highlights its potential role in herbivore-induced cotton defense. Recombinant GbTPS1 catalyzed FPP and NPP as substrates to produce selinene as the major product and the single product limonene, respectively. What is more, β-elemene and limonene showed impacts on choice behavior and growth of *H. armigera* larvae. These findings provide valuable insights into understanding induced plants defense of *G. barbadense* against chewing caterpillars and help design novel pest management strategy.

## Data Availability Statement

The names of the repository/repositories and accession number(s) can be found below: National Center for Biotechnology Information (NCBI) BioProject database under accession number: PRJNA802699.

## Author Contributions

XQH and YZ conceived and designed the experiments. HZ, EL, XZH, JK, DT, and BL performed the experiments and analyzed the data. HZ, EL, XZH, XQH, and YZ refined the manuscript. All authors read and approved the final manuscript.

## Conflict of Interest

The authors declare that the research was conducted in the absence of any commercial or financial relationships that could be construed as a potential conflict of interest.

## Publisher’s Note

All claims expressed in this article are solely those of the authors and do not necessarily represent those of their affiliated organizations, or those of the publisher, the editors and the reviewers. Any product that may be evaluated in this article, or claim that may be made by its manufacturer, is not guaranteed or endorsed by the publisher.

## Footnotes

1
http://www.geneontology.org/
