# Supplementary material for: Characterization of a Novel Insect-Induced Sesquiterpene Synthase GbTPS1 Based on the Transcriptome of Gossypium barbadense Feeding by Cotton Bollworm
Source: Front Plant Sci. 2022 Jul 13;13:898541. doi: 10.3389/fpls.2022.898541 (PMC9326391; doi:10.3389/fpls.2022.898541)
Supplement: Supplementary file 1 [file Presentation_1.zip › Supplementary Material Presentation/data sheet 1.pdf]

SUPPLEMENTARY MATERIAL

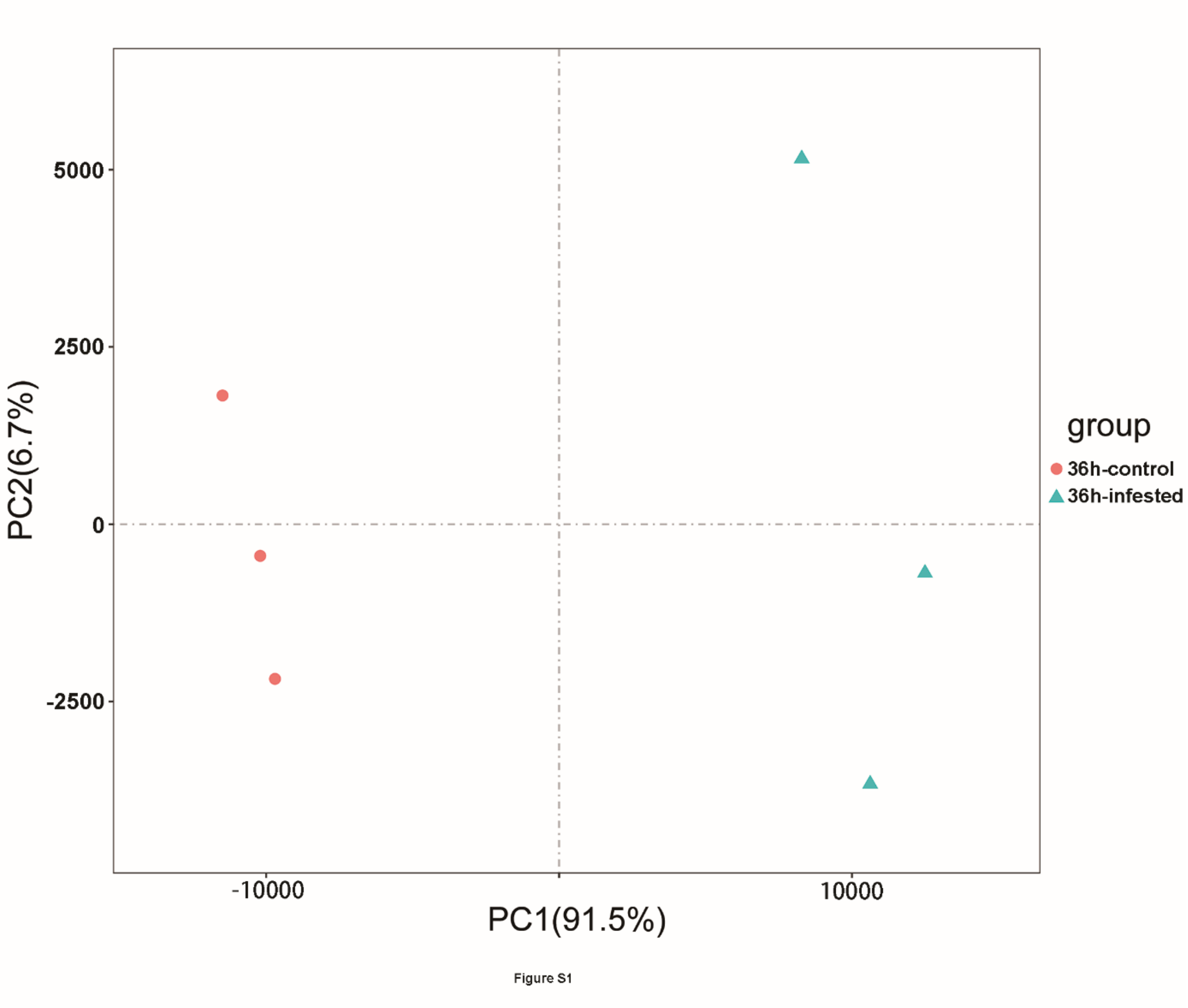

Figure S1. Principal component analysis of all genes detected in *Gossypium. barbadense* leaves infested by *Helicoverpa armigera* and non-infested controls.

Table S1 Primers used for isolation of *GbTPS1* and for qRT-PCR analysis in this study.

**Name**	**Sequence (5’→3’)**	**Usage (5’→3’)**
GbTPS1-FP	ATGCAGTGTAAAGAGGAGTGCG	isolation
GbTPS1-RP	TTAGACGGGTATTGGGTCAAG	isolation
Tubulin-QFP	GGAGCTGAGAGATTCCGTTGTC	qRT-PCR
Tubulin-QRP	TGGATTCCAGCAGCTTCCA	qRT-PCR
GbTPS1-QFP	GCCTATATGAAGCCGCCTAT	qRT-PCR
GbTPS1-QRP	GTCTTGGAGGGCATTTGTTC	qRT-PCR

GB_D06G2437-QFP	CCATCAAGGTTATCCCCGAA	qRT-PCR
GB_D06G2437-QRP	CCGTCCTTGGGTCAATATCA	qRT-PCR
GB_A09G1787-QFP	GTACTTGTTGAGCACGCATT	qRT-PCR
GB_A09G1787-QRP	GGGATTGCAGCATTTCCATT	qRT-PCR
GB_D11G0221-QFP	AGCTGTGCACGTAAAATGTC	qRT-PCR
GB_D11G0221-QRP	TGTGAAAGGGTCCAAGTGAA	qRT-PCR
GB_D06G2000-QFP	CGGGTTGCAAAGGTTTAGAG	qRT-PCR
GB_D06G2000-QRP	AATGGAAGTGGTTGCCCTAA	qRT-PCR
GB_A03G2006-QFP	TTCATTTGTCTGGTGGAGCT	qRT-PCR
GB_A03G2006-QRP	ATTTGATACCCTGGAGCCTG	qRT-PCR
GB_D08G0275-QFP	GCACTTCACCATGTTTTCGA	qRT-PCR
GB_D08G0275-QRP	CCCTAAACCAGCTGAAATGC	qRT-PCR

Table S2. All differentially expressed genes (DEGs) in *Gossypium barbadense* leaves after *Helicoverpa armigera* infestation. The DEGs were judged as differentially expressed when $\log_2|\text{Ratio}| \geq 1$ with FDR ≤ 0.05.

Table S3. Differentially expressed genes (DEGs) involved in phytohormone pathways in *Gossypium. barbadense* leaves after *Helicoverpa armigera* infestation. The DEGs were judged as differentially expressed when $\log_2|\text{Ratio}| \geq 1$ with FDR ≤ 0.05.

Table S4. *Helicoverpa armigera*-responsive transcription factors in *Gossypium barbadense* leaves. The DEGs were judged as differentiallyexpressed when $\log_2|\text{Ratio}| \geq 1$ with FDR ≤ 0.05.

Table S5. Differentially expressed genes (DEGs) involved in primary metabolism in *Gossypium barbadense* leaves after *Helicoverpa armigera* infestation. The DEGs were judged as differentially expressed when $\log_2|\text{Ratio}| \geq 1$ with FDR ≤ 0.05.

Table S6. Differentially expressed genes (DEGs) involved in secondary metabolism in *Gossypium barbadense* leaves after *Helicoverpa armigera* infestation. The DEGs were judged as differentially expressed when $\log_2|\text{Ratio}| \geq 1$ with FDR ≤ 0.05.
